# Editorial: Antiviral innate immune sensing, regulation, and viral immune evasion

**DOI:** 10.3389/fimmu.2023.1358542

**Published:** 2024-01-04

**Authors:** Wenting Lu, Ling Wang, Junji Xing

**Affiliations:** ^1^ Department of Surgery and Immunobiology and Transplant Science Center, Houston Methodist Research Institute, Houston Methodist, Houston, TX, United States; ^2^ Department of Obstetrics and Gynecology, The Second Hospital of Jilin University, Changchun, China; ^3^ Department of Cardiovascular Sciences, Houston Methodist Research Institute, Houston Methodist, Houston, TX, United States

**Keywords:** innate immunity, antiviral immunity, innate immune sensing, immune regulation, viral immune evasion, pattern recognition receptor

Antiviral innate immune response represents a critical line of defense against viral infections, encompassing sensing and responding to viruses (1, 2). Central to this defense are host pattern recognition receptors (PRRs) which play a crucial role in recognizing pathogen-associated molecular patterns (PAMPs) on the surface of viruses, mainly including Toll-like receptors (TLRs), RIG-I-like receptors (RLRs), and NOD-like receptors (NLRs) (3–6). Activation of these PRRs triggers a cascade of events, leading to the induction of cytokines, chemokines, and type I interferons (IFNs) that collectively contribute to viral clearance (7–10). Despite these formidable defense mechanisms, viruses have evolved sophisticated strategies to evade and subvert these defenses, employing a variety of mechanisms to disrupt or manipulate the host’s innate immune signaling pathways (11–13). Therefore, understanding the dynamic interplay between antiviral innate immune sensing, regulatory mechanisms, and the strategies employed by viruses for immune evasion is paramount for unraveling the complexities of host-pathogen interactions and devising innovative approaches for antiviral therapeutics.

This Research Topic “*Antiviral Innate Immune Sensing, Regulation, and Viral Immune Evasion*” highlights 47 recent studies that investigate the mechanisms about antiviral innate immune sensing and regulation in the host, and summarize the innate immune evasion strategies employed by viruses.

After a virus successfully infiltrates the host, PRRs play a crucial role in sensing the presence of viral DNAs and RNAs. Subsequently, they initiate a cascade of signal transduction events to regulate the antiviral response. Deng et al. reviewed the intricate interplay between herpesviruses and the cGAS-STING signaling pathway, a critical component of the host’s innate immunity. They discussed how herpesviruses activate or target this pathway to modulate host antiviral responses and explored potential immunotherapy strategies to boost the cGAS-STING signaling pathway. Fu et al. found that goose STING (GoSTING) played a crucial role in regulating the type I interferon pathway and contributes to the innate immune defense against RNA viruses in geese, as it induced the expression of interferons, interferon-stimulated genes, and proinflammatory cytokines while inhibiting virus replication. Li et al. investigated the regulatory role of duck laboratory of genetics and physiology 2 (duLGP2) in the duck RIG-I (duRIG-I)-mediated antiviral innate immune signaling system. They showed that duLGP2 could both suppress and enhance duRIG-I-mediated signaling pathways in response to duck Tembusu virus (DTMUV) infection, shedding light on the regulatory networks of the antiviral innate immune system in ducks. Chen et al. highlighted the involvement of RIG-I-like receptors (RLRs) and Toll-like receptors (TLRs) in the induction of type I/III interferons and ISGs, contributing to the antiviral effects of innate immunity against PEDV. The formation mechanism of membranelles organelles, known as liquid-liquid phase separation (LLPS), is not well understood in eukaryotic cells. Ye et al. examined the proteome response in Muscovy duck lung tissue during infection with highly virulent H5N1 HPAI virus (DK383) and avirulent H5N1 HPAI virus (DK212). They revealed distinct proteomic responses between the two strains, with DK383 inducing a stronger response associated with severe disease, while DK212 triggered responses related to dendritic cell maturation, phagocyte adhesion, and macrophage immune response, suggesting that these different proteome profiles, along with insights into the Akt/mTOR/p70S6K pathway, may contribute to understanding the pathogenesis of H5N1 viruses. Huang et al. identified BAG6 as a crucial negative regulator in the RIG-I-like receptor (RLR) signaling pathway. BAG6 was shown to inhibit the aggregation of the virus-induced signaling adaptor protein VISA, thereby attenuating downstream signaling by promoting K48-linked ubiquitination and inhibiting the recruitment of TRAF2, highlighting its critical role in the innate immune response to RNA virus infection. Zhang et al. examined the role of C-reactive protein (CRP) in influenza A virus infection by comparing the responses in CRP knockout mice (KO), human CRP transgenic mice (KI), and wild-type mice (WT) infected with influenza A H1N1. They showed that the absence of CRP or the presence of human CRP worsened influenza infection in mice, and CRP appeared to play a complex role in immune regulation during influenza infection. The sensing of COVID-19 virus also involves PRR activation, prompting signaling pathways to produce interferons and antiviral molecules, crucial for limiting virus replication and spread. Li et al. revealed that the SARS-CoV-2 Nsp14 protein played a role in activating NF-κB signaling, leading to the upregulation of pro-inflammatory cytokines such as IL-6 and IL-8. The interaction between Nsp14 and host Inosine-5’-monophosphate dehydrogenase 2 (IMPDH2) was identified as a crucial mechanism, and inhibiting IMPDH2 or NF-κB restricted SARS-CoV-2 infection, highlighting a potential target for therapeutic intervention. Zhaoyang et al. evaluated host DNA-removed metagenomic next-generation sequencing (mNGS) technology for detecting SARS-CoV-2 in 46 swab specimens from COVID-19 patients. The host DNA-removed mNGS demonstrated high sensitivity for detecting SARS-CoV-2, providing potential utility for comprehensive identification of the virus. Hoque et al. employed machine learning approaches to analyze RNA-seq data from COVID-19 patients, recovered individuals, and healthy individuals to identify differentially expressed genes (DEGs) and associated pathways. They found DEG signatures in both COVID-19 patients and recovered individuals, highlighting potential molecular factors and pathways connected to COVID-19 comorbidities, providing insights into the interplay between COVID-19 progression and recovery stages. Wang et al. investigated the expression of ADAM17 in normal and tumor tissues. They showed that ADAM17 expression was significantly associated with immunomodulators and immune cell infiltration, suggesting potential implications for cancer patients infected with COVID-19 and providing insights into anti-COVID-19 development strategies. Cheng et al. investigated ISG20 expression and its potential role in cancer susceptibility to SARS-CoV-2 infection. They found that ISG20 expression was elevated in various cancer types, potentially reducing vulnerability to SARS-CoV-2, and higher ISG20 expression was associated with longer overall survival in specific cancers. The signal transduction cascade culminates in the production of interferons, pivotal for orchestrating antiviral responses. Consequently, the regulation of each molecule within the signaling pathway profoundly influences the effectiveness of the antiviral response. Liu et al. investigated the role of Heat Shock Protein 90 kDa alpha class A member 1 (HSP90AA1) in classical swine fever virus (CSFV) infection. They found that overexpression of HSP90AA1 inhibited CSFV replication by interacting with the viral NS5A protein and activating JAK/STAT and NF-κB signaling pathways, providing valuable insights for potential anti-CSFV strategies. Ning et al. investigated the role of gE, a protein in the duck plague virus (DPV), by creating mutant viruses with specific gE domain deletions. They found that DPV CHv-gEΔET, a mutant with the extracellular domain of gE deleted, showed reduced virulence and could potentially be a candidate for a vaccine against duck plague. Tang et al. demonstrated that paeonol exhibited anti-virulence activity against Pseudomonas aeruginosa infection by reducing bacterial adhesion, invasion, and virulence factor expression through inhibition of quorum sensing (QS). Paeonol was also shown to enhance macrophage clearance of P. aeruginosa by modulating cytokine expression and inhibiting the TLR4/MyD88/NF-κB signaling pathway, supporting its potential as a promising anti-infective drug targeting QS and virulence factors. Ye et al. identified diagnostic markers for atherosclerosis (AS) and found 17 differentially expressed genes (DEGs) associated with AS, with FHL5, IBSP, and SCRG1 identified as potential diagnostic markers. These genes were found to be associated with various immune cells, suggesting their potential role in the development and progression of AS. Fowl adenovirus (FAdV), also known as “Angara disease,” has caused significant economic losses in the global poultry industry. Jiang et al. identified biomarkers related to rheumatoid arthritis (RA) and their connection to immune cell infiltration. Through gene analysis, they identified six hub genes, including CKS2, CSTA, and LY96, which had high diagnostic value and were associated with the concentrations of several immune cells. These findings suggested that these genes, particularly CKS2, CSTA, and LY96, could be valuable for diagnosing and treating RA. Li et al. provided opinion about the essential role of sorting nexin 5 (SNX5) in virus-induced autophagy. Ren et al. investigated the optimal concentration of Selenium Nanoparticles (SeNPs) and their mechanism in combating Porcine Delta coronavirus (PDCoV) in swine testis (ST) cells. They demonstrated that 4 μg/mL SeNPs significantly reduced PDCoV replication, alleviated PDCoV-induced mitochondrial division, and antagonized PDCoV-induced apoptosis, offering potential insights for anti-PDCoV drug development. In response to porcine epidemic diarrhea virus (PEDV) infection, LLC-PK1 cells exhibit a time- and dose-dependent upregulation of interferon-stimulated genes (ISGs), with significant activation of the JAK-STAT signaling pathway. Wei et al. reviewed the significance of LLPS in understanding viral infections and immune regulation, offering insights into potential antiviral therapeutic strategies. The heterogeneous nuclear ribonucleoproteins (hnRNPs) constitute a diverse family of RNA binding proteins with various functions in RNA metabolism, including alternative splicing, mRNA stabilization, and translational regulation. Wang et al. reviewed the roles of hnRNPs in the life cycle of positive single-stranded RNA viruses, emphasizing their interactions with viral RNA or proteins, and their regulatory effects on processes such as viral translation, genome replication, and virion release. Hu et al. investigated the effects of Tai Chi intervention on NLRP3 inflammasome and related inflammatory factors in middle-aged and older individuals with pre-diabetes mellitus (PDM). They showed that 12 weeks of Tai Chi intervention led to improvements in blood glucose, lipid levels, and insulin resistance, possibly by reducing the levels of NLRP3 inflammasome and its associated inflammatory factors in the serum of pre-diabetic patients. Lu et al. conducted whole-transcriptome sequencing on sheep embryonic testicular cells infected with Bluetongue Virus (BTV) serotype 1, and revealed 1504 differentially expressed mRNAs, 78 microRNAs, 872 long non-coding RNAs, and 59 circular RNAs, providing a more comprehensive understanding of BTV-host interactions and pathogenic mechanisms. Wang et al. established two molecular patterns of virus-related genes (VRGs) in burn patients using consensus clustering and weighted gene co-expression network analysis (WGCNA). A 2-gene signature (CD69 and SATB1) was identified as an independent prognostic factor, providing a potential biomarker for predicting survival and guiding immunotherapy strategies in burns with viral infections. Huan et al. discussed the mechanisms by which host restrictive factors inhibit enterovirus infections and highlighted the potential of those factors as targets for antiviral drug development. In sight of virus sensing and regulation, extensive vaccine development, including for COVID-19, underscores the critical need for a comprehensive understanding of host-pathogen interactions. Yuan et al. discussed the factors contributing to the low vaccine protection rate against COVID-19 and suggested that immunosuppressive parasite infections, particularly Toxoplasma gondii (T. gondii), might play a significant role in vaccine failure. Luan et al. examined the risk of antibody-dependent enhancement (ADE) in immune cell lines using immune serum from mice and humans vaccinated with alum-adjuvanted inactivated SARS-CoV-2 vaccines. These results suggested that ADE did not occur, and the lower protection rate of these vaccines might be due to lower neutralizing antibody levels or pulmonary eosinophilic immunopathology, emphasizing the need for adjustments in vaccination strategies to enhance efficacy. Ma et al. evaluated the performance of a chemiluminescent immunoassay (CLIA) for detecting specific antibodies against SARS-CoV-2 in individuals vaccinated with the Sinopharm/BBIBP vaccine. They demonstrated that high levels of neutralizing antibodies, receptor-binding-domain antibodies, and IgG persisted for over three months after the booster injection, and CLIA was consistently reliable in detecting vaccination-induced immunity. Liu et al. discussed the development of vaccines against fowl adenovirus 4 (FAdV-4) and their importance in controlling hydropericardium hepatitis syndrome (HHS), emphasizing the need for further research on cross-protection and vaccine immunogenicity. Peng et al. investigated the therapeutic potential of hepatitis B vaccine immunotherapy for occult hepatitis B virus infection (OBI) patients. They showed that hepatitis B vaccine treatment significantly increased serum hepatitis B surface antibodies, along with peripheral blood B and CD8^+^ T lymphocytes, suggesting a potential immunotherapeutic approach for OBI patients. The global health challenge posed by enterovirus infections, coupled with the lack of specific drugs and broad-spectrum vaccines, necessitates the development of effective strategies.

Despite the existence of a comprehensive and precise innate immune system designed to avoid virus infections, viruses have evolved numerous strategies to evade immune responses. Duan et al. analyzed RNA-sequencing data from COVID-19 patients and found significant changes in mitochondrion-related gene expression and functions, along with alterations in metabolic pathways. They proposed a detailed mechanism involving mitochondrial damage in COVID-19, including excessive mitochondrial fission, impaired mitochondrial degradation, and disruptions in cellular processes that contribute to immune escape and inflammation in patients. Zheng et al. utilized bioinformatics analysis on datasets related to rheumatoid arthritis (RA), Staphylococcus aureus bacteremia (SAB), and severe acute respiratory syndrome coronavirus 2 (SARS-CoV-2) to identify shared biomarkers and disease targets. The hub gene IFI44 was identified as a common factor in RA, COVID-19, and SAB, suggesting its role in immune escape mechanisms. IFI44 was shown to negatively regulate the interferon signaling pathway, promoting viral replication and bacterial proliferation, making it a potential molecular target for SARS-CoV-2 and S. aureus immune escape in RA. Jian et al. reviewed how Arter viruses employ various strategies, involving both structural and nonstructural proteins, to counteract the host’s interferon (IFN) production and impede the IFN-activated antiviral signaling pathways. Herpes simplex virus type 2 (HSV-2) can establish lifelong latency within dorsal root ganglia by evading the host’s innate immunity. Hu et al. identified the immediate early protein ICP22 of HSV-2 as a crucial viral element that inhibits NF-κB activation, demonstrating its role in suppressing host antiviral responses and providing insights into the mechanism of HSV-2 immune evasion. Liu et al. discussed the strategies employed by alphaviruses, such as CHIKV, SINV, and VEEV, to evade various components of the host antiviral innate immune response, including cGAS-STING, IFN, transcriptional host shutoff, translational host shutoff, and RNA interference (RNAi). Liu et al. found that cGAS, an innate immune DNA sensor, played a role in inhibiting porcine reproductive and respiratory syndrome virus (PRRSV) infection by sensing mitochondrial DNA (mtDNA) released into the cytoplasm during PRRSV infection. Wang et al. focused on the innate immune system of bats and identified the Tadarida brasiliensis MDA5 gene (batMDA5), which plays a major role in sensing and responding to RNA viral infections. BatMDA5 was found to activate the production of IFNβ and inhibit the replication of vesicular stomatitis virus (VSV-GFP) in bat cells, highlighting the important role of this gene in the bat’s innate immune response against RNA viruses. Zhang et al. found that both the wild-type and attenuated M6 strains of HSV-1 could infect dendritic cells and induce changes in transcriptional profiles related to innate immune and inflammatory responses. They showed that HSV-1, particularly the wild-type strain, interfered with antiviral immunity by modifying the immunological phenotype of dendritic cells, leading to deficient immune responses in infected individuals. Circular RNAs (circRNAs) as novel regulatory molecules have been recognized in diverse species, including viruses. The virus-derived circRNAs play various roles in the host biological process and the life cycle of the viruses. Zhang et al. summarized the role of circular RNAs (circRNAs) derived from both DNA and RNA viruses in host biological processes and viral life cycles. Influenza virus infection often triggers a cytokine storm, contributing to severe outcomes. Zhang et al. examined the impact of influenza infection on PPARγ expression and activity in human alveolar macrophages (AMs) and a mouse model. They demonstrated that influenza virus reduced PPARγ expression and transcriptional activity in AMs, contributing to the proinflammatory response and lung pathology associated with the infection, but PPARγ agonist treatment could mitigate these effects. Li et al. investigated the relationship between pyroptosis, a pro-inflammatory cell death process, and tumor immunity in hepatitis B virus-related hepatocellular carcinoma (HBV-HCC). They developed a pyroptosis-score (PYS) and found that higher PYS was associated with poor prognosis but increased susceptibility to anti-PD-L1 treatment in HBV-HCC patients, suggesting the potential of targeting pyroptosis as a strategy in inflammation-driven cancers. Amsden et al. summarized the IFN-dependent and IFN-independent antiviral mechanisms of IL-27 and highlighted the potential of IL-27 as a therapeutic cytokine for viral infection. Wu et al. used a duck monocyte/macrophages cell model to investigate the transcriptome associated with duck plague virus (DPV) infection. They found that DPV differentially regulated various signaling pathways, including MAPK, NF-κB, and IFN pathways, and discovered that the JNK pathway negatively regulates the IFN pathway and promotes virus proliferation. Seneca Valley virus (SVV), known for causing vesicular disease in swine, was found to inhibit the expression of Mitofusin-2 (MFN2), a protein involved in mitochondrial dynamics. Deng et al. explored the interactions between Seneca Valley virus (SVV) and host cells. They found that SVV inhibited the host protein Mitofusin-2 (MFN2), which leads to the activation of the RIG-I/IRF7 signaling pathway and increased expression of IFN-λ3, contributing to SVV’s ability to evade the host immune response and replicate. DEAD-box RNA helicase 21 (DDX21) serves as an ATP-dependent RNA helicase involved in various cellular processes, including RNA splicing, transcription, and translation. Li et al. investigated the role of DEAD-box RNA helicase 21 (DDX21) in regulating interferon production. They found that DDX21 functioned as a negative regulator of interferon-beta (IFN-β) production by competing with retinoic acid-inducible gene I (RIG-I) for binding to double-stranded RNA (dsRNA), thus helping to maintain immune homeostasis. Senecavirus A (SVA) poses a significant threat to the swine industry, causing swine vesicular disease, and its replication mechanism is not well understood. Zhao et al. found that the host protein DDX21 played a role in inhibiting Senecavirus A (SVA) replication, but SVA counteracted this antiviral effect by inducing the degradation of DDX21 through the actions of its 2B and 3C proteins, involving the caspase pathway. Tanaka et al. investigated how human metapneumovirus (HMPV) interferes with the RIG-I/TRIM25-mediated antiviral response. They found that HMPV M2-2 inhibited RIG-I signaling by forming a complex with RIG-I CARD and TRIM25, similar to the immune evasion mechanisms employed by other viruses in the Paramyxoviridae and Pneumoviridae families.

In conclusion, our gratitude goes out to all the authors who have placed their discoveries in our hands, as well as to the referees for their meticulous and perceptive evaluations. We believe that the compilation of articles in this subject area will engage researchers specializing in antiviral innate immunity and the evasion strategies employed by viruses. Understanding the intricacies of antiviral immune detection and evasion is anticipated to contribute valuable knowledge for the development of vaccines and antiviral treatments, potentially influencing the approach to the ongoing COVID-19 pandemic and other future infectious diseases.

## Author contributions

WL: Writing – original draft. LW: Writing – original draft. JX: Conceptualization, Funding acquisition, Writing – review & editing.
